# Psychological trauma in different mechanisms of traumatic injury: A hospital-based cross-sectional study

**DOI:** 10.1371/journal.pone.0242849

**Published:** 2020-11-30

**Authors:** Tulika Mehta Agarwal, Mohammed Muneer, Mohammad Asim, Malaz Awad, Yousra Afzal, Hassan Al-Thani, Ahmed Alhassan, Monira Mollazehi, Ayman El-Menyar

**Affiliations:** 1 Department of Surgery, Trauma Surgery, Hamad General Hospital, Doha, Qatar; 2 Department of Surgery, Plastic Surgery, Hamad Medical Corporation, Doha, Qatar; 3 Clinical Research, Trauma & Vascular Surgery, Department of Surgery, Hamad General Hospital, Doha, Qatar; 4 Medical Research Center, Hamad Medical Corporation, Doha, Qatar; 5 Department of Psychiatry, Hamad Medical Corporation, Doha, Qatar; 6 Department of Clinical Medicine, Weill Cornell Medical School, Doha, Qatar; Tsinghua University, CHINA

## Abstract

**Background:**

Psychological distress following traumatic injury can influence the patient health, well-being and quality of life; however, this impact may partly vary according to the type and severity of injury. We aimed to study the predominant distress causing cluster and individual symptoms of Post-Traumatic Stress Disorders (PTSD) at the clinical and subthreshold level in patients with traumatic injuries, based on the mechanism of injury (MOI).

**Methods:**

A hospital based cross-sectional study was conducted at a Level 1 Trauma Center utilizing PTSD Checklist to diagnose PTSD after one month of the traumatic event. All patients suffering from psychological distresses were assessed by a clinical psychologist in the trauma section. PTSD diagnostic criteria from DSM-5 were used to classify the patients. The inclusion criteria comprised of adult trauma patients who were directly involved in traumatic injuries and admitted under the Trauma Surgery services for a minimum of one day; have ability to provide written informed consent and can be assessed with the PCL-5 checklist after 4 weeks post-injury.

**Results:**

Two hundred patients completed PCL-5 checklist, of them 26 (13.0%) were positive for PTSD and 174 (87%) had subthreshold scores. The mean age of participants was 34.4±11.8 years and males constituted 90.5%. Road traffic injury (RTI) was most the frequent injury mechanism (59%). PTSD positive patients with RTI, fall of heavy objects, pedestrian injury and assaults had highest average scores on clusters of negative alterations in mood and cognitions (16.9, 18.0, 18.5, 17.0 respectively), followed by hyperarousal. Symptom of always being on the guard and having repeated unwanted or disturbing memories of the incident, was reported by nearly 100% PTSD positive patients. Patients with subthreshold scores also reported distressing symptoms on all four clusters of PTSD.

**Conclusions:**

Patients with different MOI showed a broad range of psychological problems with respect to symptom clusters. Negative alteration in mood and cognition followed by hyperarousal caused higher level of distress in patients post traumatic injuries. Subthreshold symptoms of PTSD are more common and deserve more attention.

## Introduction

Psychological stress following a traumatic injury can influence the patient health, well-being and quality of life. Recovery from such trauma may take a long time and is often an incomplete process. Psychological symptoms of trauma associated with traumatic injuries are often missed or discounted when patients are clinically evaluated [1–3]. Interestingly, a personal experience of trauma, witnessing a traumatic event or traumatic injury sustained by a close family member may directly or indirectly have psychological impact on the individual [4]. Such terrifying event after some time can lead to a potentially chronic and debilitating psychiatric disorder referred as post-traumatic stress disorder (PTSD) [5]. It has been included under the category of “*Trauma and Stressor-Related Disorders*” in the Diagnostic and Statistical Manual of Mental Disorders (DSM-5) [1, 6]. The primary symptoms of PTSD such as avoidance, intrusions, flashbacks, difficulty in concentration, and mood and behavioral change can jeopardize the personal and professional life of an individual [7]. Patient with subthreshold or subclinical PTSD can also be impacted by various symptoms which are associated with elevated levels of distress, impaired interpersonal and occupational functionality; however, such patients have better response to treatment as compared to those who have clinical PTSD [8].

According to the DSM-5 criteria, symptoms of PTSD can be grouped into four clusters namely intrusion or re-experiencing symptoms, avoidance and numbing symptoms, negative alterations in mood or cognitions and hyperarousal symptoms [9]. The model for clustering PTSD symptoms is found to have optimum stability across different types of traumatic injuries [10]. Association of the predominant symptoms/symptom clusters with different traumatic injuries could be useful to guide the selective psychological interventions based on the mechanisms of traumatic event.

There are many studies which have explored the prevalence and impact of PTSD in patients with traumatic injuries from the western region [11, 12]. Currently, there is a growing body of literature on trauma and PTSD in the civilian population from the Middle East region which attracts much attention [13]. Still, more studies on civilian population are needed to identify the clinical and subthreshold PTSD symptoms in patients based on the type and mechanism of traumatic injuries. A research gap has been noticed when it comes to identifying the symptoms in trauma patients that are subthreshold for PTSD. It has been observed clinically that sometimes these symptoms can cause substantial distress and have a great impact on lives of individuals who have undergone traumatic injuries. Secondly, most researches on patients with traumatic injuries have focused on studying the prevalence of clinical PTSD and the individual symptoms, however, focused studies on the cluster symptom of these patients who are subthreshold or positive for PTSD, are lacking. Such focused understanding of symptom clusters can make the psychological treatment of patients very specific for different types of injuries. This study aims to determine the predominantly distressing cluster and individual symptoms of PTSD at the clinical and subthreshold level and their relationship with different mechanism of injury (MOI) at a level 1trauma center. It is also important because it brings out the significant need for early screening and psychological care of patients who sustained traumatic injuries.

## Methods

A hospital-based cross-sectional study was conducted to include traumatic injury patients admitted at the Level 1 Trauma Center, at Hamad general hospital between September 2017 and September 2018. The inclusion criteria comprised of adult trauma patients who were directly involved in traumatic injuries and admitted for a minimum of one day; have ability to provide written informed consent with a Glasgow coma scale of 15 and can be assessed with the PCL-5 checklist after 4 weeks post-injury. All trauma patients with pre-existing mental health illness, presence of physical illnesses and psychopathologies unrelated to the accident and vulnerable population (pregnant women and children) were excluded from the study.

Utmost care has been taken to consider and avoid all sources of bias. All consents were collected by the same two coordinators who were provided appropriate training by licensed Psychologist for administering the consent and collecting the data. Telephone scripts were used by the research team while collecting the data and findings were peer-reviewed during regular team meetings. Obtained data were cross-checked against the trauma registry for ascertaining the accuracy of the injury type of each participant.

### Sample technique

The sample size was calculated to estimate a prevalence of PTSD of 15% among all admitted trauma cases with a precision of estimate (margin of error) of 5% and a 95% level of confidence. Using the single proportion equation for dichotomous variables in nMaster 2.0 sample size software package, the required sample size was 200 subjects. Additional participants were approached (n  =  120) to account for the dropout rate and loss of follow up to complete PCL-5 due to a time lag between the consent and data collection which was unavoidable as it was a requirement for administration of the diagnostic tool.

### Tool

We have used Post-Traumatic Stress Disorder Checklist (PCL-5), a structured, validated clinical interview tool based on Diagnostic Statistical Manual 5 (DSM 5) for the assessment of PTSD. Briefly, the PCL-5 checklist contains a total of twenty items rated on a five-point Likert-type scale [14]. The cutoff value of ≥33 on PCL-5 checklist was considered for the diagnosis of clinical PTSD; whereas, PCL-5 scores less than 33 was indicative of subthreshold/subclinical PTSD. This checklist is intended to assess patient symptoms in the last one-month period, which is minimum requirement for making a diagnosis of PTSD. The scale was therefore, administered at the end of one month from the date of the injury. The subclinical population that did not meet the criteria for PTSD diagnosis is often presented with symptoms which were not severe enough to present definite or readily observable symptoms.

All admitted trauma patients who were able to well communicate were approached to obtain informed consent for participation in the study. As the population in Qatar is multi-ethnic, the informed consents were available in the most common 4 languages (English, Arabic, Urdu and Hindi) and were administered in the preferred language of the subject. Subjects who agreed to participate were enrolled in the study with a written informed consent. A copy of the consent was provided to the patients to read it before and after taking the signature and they were informed that they could withdraw their consent and participation at any time without any impact of their standard of care. Patients who were unable to sign the consent due to injury to their hand or because they were illiterate, a thumb impression could be given, in the presence of a witness who could read the document in the patient language. However, final inclusion in the study was based on the completion of PCL-5 checklist at the end of one-month post-traumatic event. All those who initially provided the consent were contacted either by phone or face to face in the clinic to obtain response for the PCL-5 checklist and those who were willing to complete the PCL-5 were included in the study analysis. Trained study team members administered the PCL-5 checklist for the discharged patients. The team members, who were not from the mental health faculty and were involved in the data collection, were trained using mock sessions. This team was also audited by the mental health professional research team to ensure accuracy of data collection. Collected data were tabulated on the same day in a centralized document and re-checked for accuracy by another member of the research team. Copies of signed consents were sent to the hospital medical records department. These steps ensured quality control of the collected data.

Patients with score ≥ 33 on the PCL-5 checklist were explained about the diagnosis of PTSD and were referred to the trauma psychology service. The PTSD Symptoms were grouped into four clusters (intrusive thoughts; avoidance; negative alteration in mood or cognition and hyperarousal symptoms).

Collected data included demographics characteristics (age and gender), mechanism of injury (MOI) such as road traffic injury (motor vehicle crash, motor cycle crash, bicycle accidents), pedestrian injury, fall from height or fall while at ground level, fall of heavy objects, assault (i.e., gunshot, and stabbing) and others (self-harm, crush, explosion, machinery, blast, all-terrain vehicle and sports-related injuries), response of the PCL-5 checklist items and clustering of PTSD symptoms based on DSM-5. Definition of PTSD was given according to ICD- 9 and 10 (code 309, F43.10 and 43.11). Definitions for MOI were based on ICD- 9 codes (traffic -related injuries (E810-819), falls (E880 and E916) and assaults (E960-E969).

The study was approved by the institutional review board of the Medical Research Center at Hamad medical corporation (IRB# 14204/14). Hamad Medical Corporation is a group of governmental, non-profit hospitals in which medical services are accessible for all people free of charge in the emergency settings. All patients signed informed consent before being enrolled in the study. We did not involve the public in the study design, or conduct, or reporting, or dissemination plans of our research. This study follows the Strengthening the Reporting of Observational Studies in Epidemiology (STROBE) Statement for cross sectional studies (S1 File) [15].

### Statistical analysis

Data were presented as percentage, medians and range or mean ± standard deviation, as appropriate. A two-pronged approach was used to analyze the data which includes separate presentation of clinical (PCL-5 checklist score ≥ 33) as well as subclinical (scores <33) PTSD groups.

Analysis was carried out based on average cluster scores and independent symptom frequency for subclinical and PTSD positive population for different injury types. Microsoft Excel 2010 (Microsoft, Redmond, WA) was used to perform the data analysis.

## Results

During the study period, a total of 320 subjects consented to participate in the study, of which 120 subjects were unable to complete the PCL 5 checklist at the end of one month and dropped out. Two hundred subjects responded to the PCL-5 checklist after one month of the traumatic event and completed the study. The mean age of participants was 34.4±11.8 years; and 90.5% were males. Road traffic injury (RTI) including accidents of motorized vehicles as well as non-motorized vehicles like bicycles (59.0%) was the most frequent MOI, followed by fall from height (20%), pedestrian injury (8%), fall of heavy object (7.5%), other injuries (3.5%) and assault (2%) (Table 1).

**Table 1:** Distribution of PTSD and subclinical PTSD by injury mechanisms.

	Road traffic injury*	Pedestrian	Fall from height	Fall of heavy object	Assault	Others	P value
	[n = 118 (59.0%)]	[n = 16 (8.0%)]	[n = 40 (20.0%)]	[n = 15 (7.5%)]	[n = 4 (2.0%)]	[n = 7 (3.5%)]	
**Subclinical PTSD (n = 174)**	100 (84.7%)	14 (87.5%)	37 (92.5%)	14 (93.3%)	2 (50.0%)	7 (100.0%)	0.15
**Clinical PTSD (n = 26)**	18 (15.3%)	2 (12.5%)	3 (7.5%)	1 (6.7%)	2 (50.0%)	0 (0.0%)	

* motor vehicle crash, bike and motorcycle crash.

Of the 200 subjects who were exposed to traumatic events, 26 (13.0%) patients met PCL-5 criteria for PTSD (score ≥ 33) and the remaining 174 (87%) had subthreshold PTSD scores when assessed after one month of the injury (Table 2). Patients who had experienced different types of traumatic injuries showed marked variability in the frequency of developing PTSD. Fivety percent of the assault victims, 12.5% of pedestrian injury patients and 15.3% victims of the RTI had developed PTSD, while 7.5% patients with injuries related to fall and 6.7% patients after fall of heavy object showed relatively lower frequency of PTSD.

**Table 2:** Average cluster scores for PTSD positive and sub-threshold PTSD cases.

	Subclinical PTSD (n = 174)	PTSD (n = 26)	P value
**PCL-5 subscales**	10.17±8.52	44.35±8.29	0.001
**Intrusion (Q1-Q5)**	2.14±2.49	9.54±3.87	0.001
**Avoidance (Q6-Q7)**	1.36±2.02	5.42±2.19	0.001
**Cognition and mood (Q8-Q14)**	3.71±4.14	16.73±4.45	0.001
**Arousal and reactivity (Q15-Q20)**	2.98±2.66	12.65±3.81	0.001

Patients with RTI, pedestrian injuries, fall of heavy objects, and assault scored highest on cluster of negative alteration in mood/cognition, followed by hyperarousal. Patient with fall from height related injuries scored highest on cluster of hyperarousal followed closely by negative alteration in mood/cognition (Table 3).

**Table 3:** Average cluster scores for cases with PTSD and sub-threshold PTSD according to mechanism of injury.

	Intrusive thoughts		Avoidance		Negative alteration in mood or cognition		Hyperarousal	
	PTSD	Subclinical PTSD	PTSD	Subclinical PTSD	PTSD	Subclinical PTSD	PTSD	Subclinical PTSD
**Road traffic injury**	9.33	2.03	5.28	1.45	16.94	4.31	11.89	3.33
**Pedestrian**	8.50	2.93	6.00	1.29	18.50	3.64	12.50	3.07
**Fall from height**	10.00	2.08	6.67	1.27	13.67	2.70	14.00	2.51
**Fall of heavy object**	11.00	2.21	5.00	1.00	18.00	1.86	16.00	2.21
**Assault**	11.00	3.50	4.50	1.50	17.00	9.0	16.00	1.50
**Others**	0.00	2.00	0.00	1.29	0.00	2.71	0.00	2.29

### PTSD positive patients with RTI

When individual symptoms were analyzed, it was found that many PTSD positive patients experienced intrusive thoughts such as repeated disturbing memories of the incident, nightmares, felt upset upon being reminded, avoided memories, feelings and external reminders associated to the incident. They often had difficulty in remembering important parts of the incident and had strong negative feelings such as blame, guilt, fear, anger, associated with it. Many of these patients experienced loss of interest, felt cut off from people around them and had difficulty in experiencing positive feelings. They were hyperalert, got startled easily, and had sleep- related difficulties (Table 4).

**Table 4:** Scores of clinically positive PTSD patients.

PCL5 questionnaire	RTI (n = 18)	Pedestrian (n = 2)	Fall from height (n = 3)	Fall of heavy object (n = 1)	Assault (n = 2)
**1. Repeated, disturbing and unwanted memories of the stressful experience?**	77.8%	100%	100%	100%	100%
**2. Repeated, disturbing dreams of the stressful experience?**	61.1%	50.0%	66.7%	100%	50.0%
**3. Suddenly feeling or acting as if the stressfull experience were actually happening again (as if you were actually back there relieving it)?**	50.0%	0.0%	100%	100%	50.0%
**4. Feeling very upset when something remind you of the stressful experience?**	83.3%	100%	66.7%	100%	100%
**5. Having strong physical reactions when something reminded you of the stressful experience (for example, heart poundig, trouble breathing, sweating)?**	61.1%	50.0%	0.0%	100%	50.0%
**6. Avoiding memories, thoughts, or feelings related to the stressful experience?**	94.4%	100%	66.7%	100%	100%
**7. Avoiding external reminders of the stressful experience for example (people, places, conversations, activities, objects, or situations)?**	83.3%	50.0%	100%	100%	50.0%
**8. Trouble remembering important part of the stressful experience?**	72.2%	50.0%	0.0%	100%	0.0%
**9. Having strong negative believes about yourself, other people, or the world (for example having thoughts such as, i am bad there is something seriously wrong with me, no one can be trusted, the world is completely dangerous)?**	50.0%	100%	66.7%	100%	50.0%
**10. Blaming yourself or someone else for the stressful experience or what happened after it?**	83.3%	100%	100%	100%	100%
**11. Having strong negative feelings such as fear, horror, anger, guilt, or shame?**	94.4%	100%	100%	100%	100%
**12. Loss of interest in activities that you used to enjoy?**	78.8%	50.0%	33.3%	100%	50.0%
**13. Feeling distant or cut off from other people?**	77.8%	50.0%	66.7%	100%	100%
**14. Trouble experiencing positive feelings (for example, being unable to feel happiness or have loving feelings for people close to you)?**	83.3%	100%	66.7%	100%	100%
**15. Irritable behavior, angry outbursts, or acting aggressively?**	61.1%	50.0%	100%	100%	100%
**16. Taking too many risks or doing things that could cause you harm?**	11.1%	0.0%	0.0%	0.0%	0.0%
**17. Being “superalert” or watchful or on guard?**	88.9%	100%	100%	100%	100%
**18. Feeling jumpy or easily startled?**	77.8%	50.0%	66.7%	100%	100%
**19. Having difficulty concentrating?**	66.7%	50.0%	33.3%	100%	100%
**20. Trouble falling or staying asleep?**	77.8%	100%	100%	100%	100%

### PTSD positive patients of pedestrian injury/ or assault related injuries

These patients reported difficulties due to intrusive thoughts especially with repeated disturbing and unwanted memories of the stressful event and felt upset by reminders of the stressful experience. They tended to avoid memories or thought related to the incident, experienced strong feelings of blame for self or others, and had strong negative feelings such as fear, horror or guilt. Both groups had difficulty in experiencing positive feelings. They were hyperalert and watchful and reported difficulty in sleeping well.

Assaulted patients also reported being more irritable and aggressive, easily startled and suffering from lack of concentration.

Pedestrian injury patients, on the other hand, suffered strong negative belief about self and people around them (Table 4).

### Positive PTSD patients with fall related injuries

Patients with positive PTSD and fall related injuries showed a slight deviation from other groups and scored highest average scores for hyperarousal followed by negative alteration in mood and cognition, intrusive thoughts, flashbacks but did not experience any related physical sensation upon being reminded. They showed avoidance towards any reminders of the stressful experience. They were more likely to blame self or others for what happened and harbored strong negative feelings in their mind. These patients experienced anger outbursts and easily acted aggressive, were super alert and faced sleep related difficulties.

Patients with injuries such as all-terrain vehicle injury, explosion, machinery, crush or sports related injuries were categorized under other injuries. All these patients experienced intrusive thoughts, felt upset by reminders of the incident, avoided memories and thoughts, blamed self or others, harbored strong negative feelings such a fear, guilt, horror, etc. They often got angry, irritable or behaved aggressively, were hyper alert, had difficulty in concentrating, staying asleep and easily got startled.

### Subclinical *PTSD* population scores

For the subclinical population, average impact on cluster scores varied based on the type of injury. Patients who had undergone RTI, pedestrian injury or a fall from height had highest average score for altered emotional response followed by hyperarousal (Table 3). However, patients of fall of heavy objects scored average high on both hyperarousal and intrusive thoughts. Patients, who were involved in an assault, primarily scored average high on negative alteration in mood and cognitions followed by intrusive thoughts.

Table 5 shows that subsets of traumatic injury patients who had subthreshold scores on PCL5 also experienced some psychological symptoms. Several patients suffered components of intrusion such as suffering from distressing intrusive memories of the stressful experience (50% RTI, pedestrian injuries, and assault). Many patients with subthreshold scores were likely to feel very upset when something reminded them of the stressful experience (victims of pedestrian injuries 79% fall of heavy object 57% and assault 50%). Often patients of RTI and assault had difficulty in remembering important parts of the event (RTI 40%, Assault 50%) and blamed themselves or others for what happened (RTI 41%, Assault 50%). Blame was also common amongst patients of pedestrian injuries (43%). Higher proportion of patients indicated distress due to increased watchfulness (93% pedestrian injury patients, 65% RTI, 57% fall of heavy object, 57% fall from height and 71% other types of injuries) and sleep-related difficulty (pedestrian 50%, and assault 50%).

**Table 5:** Scores of subthreshold patients for different symptoms of PTSD.

PCL5 questionnaire	RTI (n = 100)	Pedestrian (n = 14)	Fall from height (n = 37)	Fall of heavy object (n = 14)	Assault (n = 2)	Others (n = 7)
**1. Repeated, disturbing and unwanted memories of the stressful experience?**	50.0%	50.0%	45.9%	42.9%	50.0%	42.9%
**2. Repeated, disturbing dreams of the stressful experience?**	6.0%	21.4%	5.4%	7.1%	0.0%	0.0%
**3. Suddenly feeling or acting as if the stressfull experience were actually happening again (as if you were actually back there relieving it)?**	11.0%	7.1%	18.9%	7.1%	0.0%	14.3%
**4. Feeling very upset when something remind you of the stressful experience?**	34.0%	78.6%	29.7%	57.1%	50.0%	57.1%
**5. Having strong physical reactions when something reminded you of the stressful experience (for example, heart poundig, trouble breathing, sweating)?**	12.0%	28.6%	13.5%	21.4%	50.0%	0.0%
**6. Avoiding memories, thoughts, or feelings related to the stressful experience?**	30.0%	35.7%	24.3%	35.7%	50.0%	42.9%
**7. Avoiding external reminders of the stressful experience for example (people, places, conversations, activities, objects, or situations)?**	29.0%	35.7%	24.3%	21.4%	50.0%	28.6%
**8. Trouble remembering important part of the stressful experience?**	40.0%	14.3%	24.3%	21.4%	50.0%	42.9%
**9. Having strong negative believes about yourself, other people, or the world (for example having thoughts such as, i am bad there is something seriously wrong with me, no one can be trusted, the world is completely dangerous)?**	13.0%	28.6%	21.6%	7.1%	50.0%	28.6%
**10. Blaming yourself or someone else for the stressful experience or what happened after it?**	41.0%	42.9%	16.2%	7.1%	50.0%	14.3%
**11. Having strong negative feelings such as fear, horror, anger, guilt, or shame?**	24.0%	28.6%	16.2%	35.7%	50.0%	14.3%
**12. Loss of interest in activities that you used to enjoy?**	16.0%	21.4%	8.1%	21.4%	50.0%	28.6%
**13. Feeling distant or cut off from other people?**	13.0%	14.3%	10.8%	14.3%	0.0%	0.0%
**14. Trouble experiencing positive feelings (for example, being unable to feel happiness or have loving feelings for people close to you)?**	20.0%	28.6%	24.3%	14.3%	0.0%	14.3%
**15. Irritable behavior, angry outbursts, or acting aggressively?**	15.0%	0.0%	10.8%	7.1%	50.0%	14.3%
**16. Taking too many risks or doing things that could cause you harm?**	11.0%	0.0%	0.0%	0.0%	0.0%	0.0%
**17. Being “superalert” or watchful or on guard?**	65.0%	92.9%	56.8%	57.1%	0.0%	71.4%
**18. Feeling jumpy or easily startled?**	13.0%	0.0%	5.4%	14.3%	0.0%	0.0%
**19. Having difficulty concentrating?**	8.0%	0.0%	8.1%	0.0%	0.0%	0.0%
**20. Trouble falling or staying asleep?**	23.0%	50.0%	18.9%	21.4%	50.0%	28.6%

## Discussion

This is the first study from the Arab Middle East that describes the impact of different MOI on the development of PTSD in its subthreshold or clinical form. The body’s failure to return to its pre-traumatic state differentiates PTSD from a simple fear response. Approximately 25 to 30 percent of victims of traumatic injuries resulting from RTI develop symptoms of PTSD; however, response to trauma varies with the severity and subjective experience associated with trauma [16, 17]. In the present study, although subthreshold PTSD was more common, it was found that 13% patients of traumatic injuries developed PTSD when assessed after one month of the traumatic event. A high number of patients who did not meet the cutoff for being diagnosed positive for PTSD (≥33 on PCL-5) reported that the last experienced incident was the most traumatic event in their life and they reported at least one symptom of the four clusters (Criteria B, C, D and E) after one month of the incident (Criterion F), which was indicative of a subthreshold syndrome. It was observed that though cluster scores were comparatively lower in the subthreshold PTSD population, however, distress was indicated by many of them for several symptoms that persisted with respect to different types of traumatic injuries. Therefore, it becomes important to identify patients who might be experiencing subthreshold symptoms and to provide them with the appropriate psychological care as early as possible during their hospital admission.

The impact of trauma on cluster of negative emotional alteration and hyperarousal have been frequently reported by our PTSD positive population as well as subthreshold population for all types of injuries and similar results have been reported in other studies [18–20]. Murray et al., demonstrated that continuous dissociation and thoughtfulness after one months of a motor vehicle crash (MVC) helps in recognizing patients who are at high-risk of developing PTSD [21]. The authors reported disturbing memories, thoughts, stressful experience and feeling jumpy or easily startled as the frequent identified severe symptoms.

In the present study, PTSD in victims of RTI was found to be lower (15.3%) after one month of the injury when compared to earlier reports which suggested the occurrence of PTSD in 16.71%-33% of MVC victims [16, 22]. The occurrence of PTSD was also low for patients with pedestrian injury (12.5%). A recent longitudinal study included 299 adult car accident survivors (pedestrians 27.8%) and showed a high PTSD prevalence of 46.5% [23]. One probable reason for the lower development of PTSD in the present study, is the establishment of a Trauma psychology service in the section of Trauma Surgery, which started screening and providing early psychological care for all traumatic injury patients from the time of admission. As per research evidence, early psychological interventions reduce the risk of developing PTSD in trauma patients [24].

Most clinical PTSD as well as many subthreshold patients in our study reported intrusive memories of the stressful experience post-injury, which was more distressing for PTSD positive patients. A study conducted by Hayes et al., brought out a neurobiological basis for the characteristic strong trauma memories in PTSD patients and the associations that are formed during trauma with the sensory cues that individuals experienced right before or during their traumatic event, which later serve as triggers for intrusive memories [25].

All PTSD patients irrespective of the type of injury experienced strong negative feelings of fear, anger, guilt or shame. Such negative feelings have been explained in emotional theory concerning psychological trauma. The response of fear is peri-traumatic, starting at the time of the event while guilt, anger or shame are post-traumatic symptoms of the incident that happen as a result of appraisal of the ones behavior, oneself or the external agent in the incident [26, 27]. Since the PCL-5 tool is administered after one month of the incident, these negative feelings of patients associated with the traumatic incidents could be adequately captured.

Patients with clinical PTSD as well as those who had been in an RTI or assault related trauma with a subthreshold PTSD score, blamed self or others for what happened. This self-blame could be resulting from one’s perception of avoidability for which the individual tended to self-implicate [28].

During this study period, no cases of sexual assaults were reported however, out of the four cases of physical assault that were reported, two developed PTSD. As reported by a systematic review, the prevalence of PTSD increases over time from in case of intentional trauma [20]. Therefore, there is a need to conduct a longitudinal study to address the development of PTSD post-assault injury in our community. It is important to note that in the present study, PTSD after assault showed a higher impact on the four cluster symptoms of intrusion, avoidance, negative mood alterations and arousal. Our finding is in agreement with a recent systematic review focused on PTSD following violence-related injury and reported a wide range of prevalence [29]. The current literature suggests that assault victims are at higher risk of developing psychological illness and PTSD [30]. In our cohort, the PTSD assault victims showed moderate to high irritability, experienced anger outburst or aggressive behavior since the incident happened, and they were also extremely alert or watchful, startled very easily, having moderate to high level of difficulty in concentrating and had sleeping difficulties. These difficulties which are related to numbing and hyperarousal are indicative symptoms of PTSD or depression [31]. Trouble in falling asleep or staying asleep was a common problem reported by almost all PTSD patients and 50% of patients with subthreshold scores who sustained pedestrian injury or assault. In an earlier study that investigated sleep dysfunction and physiological responses to stress, the authors reported an association between poor sleep quality, hypertension and PTSD symptoms [32]. Sleep disturbance has been identified as a core symptom of PTSD by several researchers [33, 34]. Therefore, there is a strong need to address sleep deprivation and cognitive impairment early in trauma patients.

PTSD patients who had fall related injuries suffered intrusive memories and felt extremely upset (hyperarousal) when something reminded them of the stressful experience. Also, nearly half of patients had experienced such symptoms in the subthreshold population. A study on fall-related injury in elderly patients concurred with the findings that hyperarousal is a predominant symptom of PTSD [35]. Emotional disturbance upon being reminded about the incident of fall was documented by 67% of our fall related injuries patients, a finding that was reported previously [36].

Only one patient of the 15 who suffered injury due to fall of heavy object developed PTSD. These injuries were mostly work related. Therefore, the results were not found to be conclusive. However, in the subthreshold population, high number of individuals reported distress due to hypervigilance and felt upset upon being reminded of the incident. Pain is an important contributing factor to these symptoms which correlates with lowering of mood, increasing anxiety, irritability and tendency for catastrophizing [36, 37].

A recent study on athletes reported that symptoms of mental illness post trauma such as avoidance, hypervigilance and dissociative behaviors may leads to delayed recovery from injuries and could profoundly affects sports performance outcomes [38]. The current study had one patient with sport-related injury who was not found to have clinical PTSD, however, he had symptoms suggestive of negative alteration in emotion.

## Limitations

The observations of the present study must carefully be considered in the light of certain limitations. First, the sample size yielded relatively smaller subset in each injury mechanism thereby reducing the generalizability of the findings. Also, we could not get the completed PCL-5 from the drop-out patients. Selection bias cannot be ignored as we missed the mild trauma cases that were seen and sent home from the emergency room or presented to other primary healthcare facilities. Also, only patients who consented to participate in the study were approached for data collection. Thus, the estimated prevalence of PTSD and its subclinical form in our trauma population needs further elaboration. Second, we lack PTSD assessments on follow-up of 6- and 12-month and so we cannot comment on the delayed onset of PTSD symptoms in our cohort. Third, there is possibility of selection bias due to refusal of some subject to participate in a research investigation. The results showed a high number of subthreshold PTSD. However, this study may underscore subthreshold PTSD which fulfill Criterion F that should be regarded as specific nosological categories or as specified PTSD subcategories, i.e. subsyndromal or partial PTSD. However, our Qatar national trauma data have internal and external regular validation since the data are obtained from the only tertiary level 1 trauma center in the country and connected with the national trauma databank (NTDB) in the USA; therefore, the findings provide valuable information on the hospitalized PTSD in the country.

## Conclusions

The present study highlighted the importance of considering subthreshold PTSD as manifestation of psychological distress which needs to be addressed appropriately and early during the hospitalization of trauma patients regardless of the MOI. PTSD positive patients who sustain traumatic injuries undergo marked psychological distress which should also be taken into consideration early, together with the clinical management. Negative alteration in mood and cognition and hyperarousal are the most significant clusters of impact for all types of traumatic injuries that were taken into consideration in the present cohort. Patients with different injury mechanisms showed a broad range of psychological problems with respect to symptom clusters. Therefore, a customized psychological treatment plan should be formulated based on the valence of the impacted cluster in trauma patients. In addition, a longitudinal study to assess the variability in PTSD symptoms on follow-up is warranted to better understand the need for psychological interventions in trauma patients.

## Supporting information

S1 File STROBE statement—checklist of items that should be included in reports of observational studies. (DOCX)
